# Gradual exposure to Coriolis force induces sensorimotor adaptation with no change in peripersonal space

**DOI:** 10.1038/s41598-022-04961-1

**Published:** 2022-01-18

**Authors:** Nicolas X. Leclere, Fabrice R. Sarlegna, Yann Coello, Christophe Bourdin

**Affiliations:** 1 Aix Marseille Univ, CNRS, ISM, Marseille, France; 2 Univ. Lille, CNRS, Lille, UMR 9193 - SCALab - Sciences Cognitives et Sciences Affectives, Lille, France

**Keywords:** Cognitive neuroscience, Perception, Human behaviour, Motor control

## Abstract

The space immediately surrounding the body is crucial for the organization of voluntary motor actions and seems to be functionally represented in the brain according to motor capacities. However, despite extensive research, little is known about how the representation of peripersonal space is adjusted to new action capacities. Abrupt exposure to a new force field has been shown to cause the representation of peripersonal space to shrink, possibly reflecting a conservative spatial strategy triggered by consciously-perceived motor errors. The present study assessed whether the representation of peripersonal space is influenced by gradual exposure of reaching movements to a new force field, produced by a stepwise acceleration of a rotating platform. We hypothesized that such gradual exposure would induce progressive sensorimotor adaptation to motor errors, albeit too small to be consciously perceived. In contrast, we hypothesized that reachability judgments, used as a proxy of peripersonal space representation, would not be significantly affected. Results showed that gradual exposure to Coriolis force produced a systematic after-effect on reaching movements but no significant change in reachability judgments. We speculate that the conscious experience of large motor errors may influence the updating of the representation of peripersonal space.

## Introduction

In fighting sports such as boxing, perception of the space separating a boxer from his/her opponent is critical, to avoid being hit or to throw an efficient punch as soon as an opportunity arises. This space in which a motor action can take place, for individual or social purposes, is commonly defined as the peripersonal space^1^ and can be conceived as the functional representation of the space at reach^2,3^. Stimuli presented in the peripersonal space activate brain areas differently from stimuli presented in the more distant extrapersonal space, in particular in the motor, pre-motor and parietal cortices^4–7^. In fact, the brain areas underlying the perception of objects in peripersonal space partially overlap with the brain areas underlying voluntary motor action and motor imagery^8,9^. This is consistent with the current view that the representation of peripersonal space involves both perceptual and motor components^10–16^.

Several studies have highlighted the plastic nature of the representation of peripersonal space^17,18^. For instance, the representation of the peripersonal space was found to change after the short-term use of a tool^19–21^, or following sensorimotor adaptation to a visuo-spatial perturbation^22–25^. For instance, Bourgeois and Coello^23^ studied the effect of a visuomotor perturbation on the representation of the peripersonal space by introducing a gain change, i.e., a geometrical change in the relation between the amplitude of a targeted arm movement and its seen spatial consequences. They observed an adaptation of the sensorimotor control processes as well as a change in the reachability judgments, a proxy of the representation of the peripersonal space. Moreover, the change in reachability judgments was governed by the geometrical gain, with the representation of the peripersonal space shrinking when the geometrical gain increased, and vice-versa.

Leclere et al.^26^ further studied the plasticity of the representation of the peripersonal space by assessing how it would change when the gravito-inertial force field changes. In their study, participants were seated on a platform whose rotation produced an altered force field perturbing, via the Coriolis force, the natural trajectory of arm movements toward a visual target. Abrupt exposure to the new force field was associated with a systematic change in sensorimotor control, confirming previous studies^27–32^, but also with a change in reachability judgments. More specifically, Coriolis forces perturbed rightward the straight-ahead movements, and a leftward sensorimotor adaptation was observed as well as a leftward shift of the reachability judgments. Two control experiments revealed that the modification of the representation of the peripersonal space was not due to the platform rotation or to the repetition of reaching movements per se^26^. Leclere et al.^33^ later provided evidence of a direction-specific adaptation of the sensorimotor system^34,35^, revealed by a systematic reduction of reaching errors during the exposure to the new gravito-inertial force field and, crucially, direction-specific after-effects. In contrast, direction-specific changes were not observed on reachability judgments, as a systematic contraction of the representation of the peripersonal space was found irrespective of the direction of the altered force field and resulting sensorimotor adaptation.

These recent results suggest that an abrupt perturbation of limb dynamics triggers changes in sensorimotor control processes and in the representation of the peripersonal space, but in specific and distinct ways. One possibility is that the distinct effects of opposite force fields are specifically linked to distinct sensorimotor and cognitive processes underlying arm reaching and reachability judgments, respectively. The fact that in Leclere et al.^33^, the representation of the peripersonal space was not modified in the direction predicted from the sensorimotor adaptation was consistent with an overall conservative strategy, which may result from the conscious perception of large motor errors induced by the abrupt and substantial change in the force field. However, sensorimotor adaptation does not necessarily imply large, consciously detected, motor errors. Gradually-introduced perturbations have also been shown to produce sensorimotor adaptation, to an extent relatively similar to abruptly-introduced perturbations^36,37^, even though the resulting motor errors remained small and hardly detectable at the conscious level^23,38–41^. For both types of perturbations, the perceived difference between predicted and actual sensory consequences of motor commands, i.e., the sensory prediction error, is assumed to drive the trial-by-trial updating of the internal model of limb dynamics, progressively adapting motor commands and associated sensory predictions to the new dynamic context^42–45^. An experimental landmark of this adaptation is the large error of the goal-directed movement, the so-called after-effect of the adaptation, which is observed in the opposite direction of the altered force field as soon as the exposure to it ends^29,46,47^.

While sensorimotor adaptation to a gradually-modified force field has been widely explored using robotic devices^48–52^, no study has yet investigated, to our knowledge, how the sensorimotor system adapts to a gradually-increased force field induced by a platform rotation. The consequence of such perturbation on the representation of the peripersonal space therefore also remains unknown. The present study tested whether adaptation to gradual exposure to a new gravito-inertial force field also influences the representation of the peripersonal space. Assuming that cognitive factors associated with the conscious detection of large motor errors may influence the plasticity of the representation of the peripersonal space, we hypothesized that a gradual increase in Coriolis force, which presumably results in sub-conscious motor errors, influences the action control system but only marginally influences the representation of the peripersonal space.

To test this hypothesis, we asked adult participants, seated on a rotating platform, to reach toward a visual target while the platform rotated at a gradually increased velocity, so as to incrementally increase the strength of the Coriolis force. The representation of the peripersonal space was assessed through reachability judgements before and after the exposure to a gradual change in the gravito-inertial force field. Therefore, reachability judgments were obtained before and after the action control system was updated as in our previous experiments^26,33^. Considering that previous studies reported that adaptation to gradually-altered limb dynamics also induces after-effects^36,50,52,53^, we predicted that exposure to gradual change in the Coriolis force would result in sensorimotor adaptation revealed by post-rotation after-effects on reaching movements, as typically observed after exposure to an abrupt change in Coriolis force. As mentioned before, such change typically influences the representation of the peripersonal space^26,33^, which was predicted here to not significantly differ between the pre-gradual rotation and the post-gradual rotation phases.

## Materials and methods

### Participants

Fifteen healthy right-handed adults (seven females, eight males; mean age = 21.4 ± 2.8 years) participated in this experiment. Participants gave their written informed consent prior to being included in the study, which was approved by the institutional review board of the Institute of Movement Sciences and was performed in accordance with the ethical standards set out in the 1964 Declaration of Helsinki. All participants had normal or corrected-to-normal vision and were naïve as to the purpose of the experiment.

### Experimental set-up

**Figure 1:**
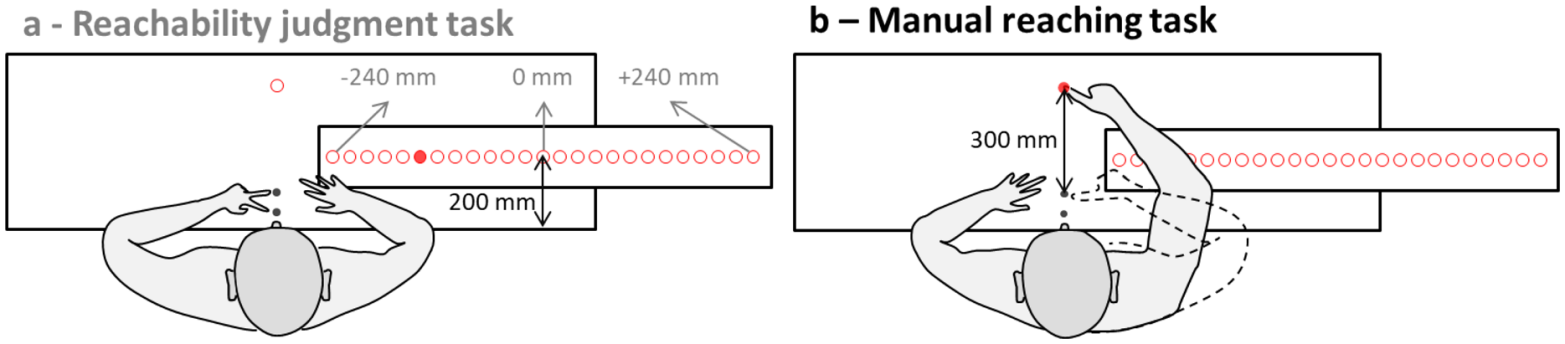
Experimental setup. (**a**) Reachability judgment task: participants had to judge whether a target illuminated on their right side was reachable or not, pressing the closer response button with their left index for “reachable” or the more distant button with their middle finger for “unreachable”. The 0 mm target position was physically adjusted for each participant to correspond to the maximum physical distance reachable with the arm fully stretched. (**b**) Manual reaching task: participants had to reach the visual target with their right index as accurately and as fast as possible.

The experimental set-up was identical to that used in our previous studies^26,33^. Participants sat at the centre of a motorised rotating platform. An adjustable headrest was used to restrain head movements and to keep the centre of the head aligned with the vertical axis of the platform, so as to minimize centrifugal forces applied on the head during platform rotation^26–32^. We used a rotating platform so that when the upper limb was voluntarily moved toward the target during rotation, each point of the limb moving out of the center of rotation was subjected to the Coriolis force (F_cor_ in the following equation) acting perpendicularly to the limb displacement: F_cor_ = − 2 m × ω × v, with m the mass of the upper-limb segments in motion, ω the platform’s angular velocity and v the arm’s linear velocity^27^. Centrifugal force was thought to be negligible, as in previous work ^26,27,29,31^. As Fig. 1 shows, several visual targets were positioned on a horizontal table placed in front of the participants, at waist level. All visual targets were low-intensity red light-emitting diodes (3 mm in diameter) presented in an otherwise completely dark room.

Participants had to perform two tasks, each of them involving different visual targets^26,33^. For the manual reaching task, the visual target was located 30 cm from the starting hand position along the mid-body sagittal axis (Fig. 1). Participants had to reach with the index fingertip toward this visual target: the fingertip motion was thus mainly in the sagittal plane. Considering the characteristics of the Coriolis force described earlier, the platform rotation produced a Coriolis force which perturbed the reaching movement mainly along the frontal plane. Thus, sensorimotor adaptation to the Coriolis was expected mainly along the frontal plane. This led us to use, for the reachability judgment task, 25 visual targets which were aligned horizontally in the participants’ right hemispace (perpendicularly to the reach movement in the mid-body sagittal axis and according to the direction of the perturbation induced by the Coriolis force). The reachability targets were located, for each individual, between ± 240 mm (inter-target distance 20 mm) of the maximum arm length (see Fig. 1 and Procedure). On the horizontal table, two response buttons were positioned close to the participant, one located 1 cm from the table’s proximal edge and the other located 1 cm farther away. Participants operated these buttons with their left hand to respond in the reachability judgment task (near button for responding ‘reachable’ and far button for responding ‘non-reachable’ after a given was illuminated). The far button in the reachability judgment task also served as the starting position for the right hand in the manual reaching task, and could be illuminated with a light-emitting diode.

An infrared active marker was taped to the right index fingertip, whose position was sampled at 350 Hz using an optical motion tracking system (Codamotion cx1 and MiniHub, Charnwood Dynamics Ltd, Leicestershire, UK), to record hand movement kinematics during the manual reaching task. Response buttons were sampled at 800 Hz to record reachability judgments. The experimenter controlled the tracking system, the motorised platform and the presentation of the visual targets from an adjacent room via customised software (Docometre) governing a real-time acquisition system ADwin-Pro (Jäger, Germany).

### Procedure

The procedure was identical to that used in our previous studies^26,33^ except for the gradual introduction of the platform rotation. Once seated on the platform and before the experiment started, participants wore occluding glasses to prevent them from viewing the target array. They were then asked to fully stretch out their right arm in the fronto-parallel plane: this allowed the experimenter to match the position of each participant’s index fingertip, arm fully stretched, with the position of the central target in the array used for the reachability judgment task. The individually-adjusted position of the central target thus corresponded to the actual maximum distance that was physically reachable by each participant^21,26,54,55^. After this personalized adjustment of the setup, the occluding glasses were removed and participants were allowed to open their eyes in the dark room.

#### Manual reaching task

In the manual reaching task, each trial began with the right index positioned at the starting hand location. The visual target was flashed for 200 ms after a 100 ms auditory tone, followed by a random period of 500–1000 ms. As soon as the visual target was turned on, participants had to reach toward it as fast and accurately as possible with the right index. The visual target was covered by a plexiglass plate and neither tactile nor visual feedback was available to participants. These were asked to maintain their final hand position once the finger touched the horizontal board. 3.5 s after the start of the reaching movement, the LED at starting hand location was turned on: this indicated the end of the trial and signaled to participants that they should move their hand back to the start position and prepare for the next trial. No explicit instructions were given with respect to hand path.

#### Reachability judgment task

In the reachability judgment task, after a 100 ms auditory tone followed by a random period of 500–1000 ms, one of the 25 visual targets was randomly presented in the participants’ right hemispace. Participants had to judge as fast and accurately as possible, without performing any reaching movement, whether the illuminated visual target was reachable or not with their right index, considering a stable trunk posture. This two-alternative forced choice was recorded as participants pressed either the near response button (“reachable”) with their left index or the far response button (“unreachable”) with their middle finger. The target disappeared as soon as participants provided their response and, at the end of a fixed period lasting 4 s from the 100 ms auditory tone, the next trial started with the same temporal sequence.

**Figure 2:**
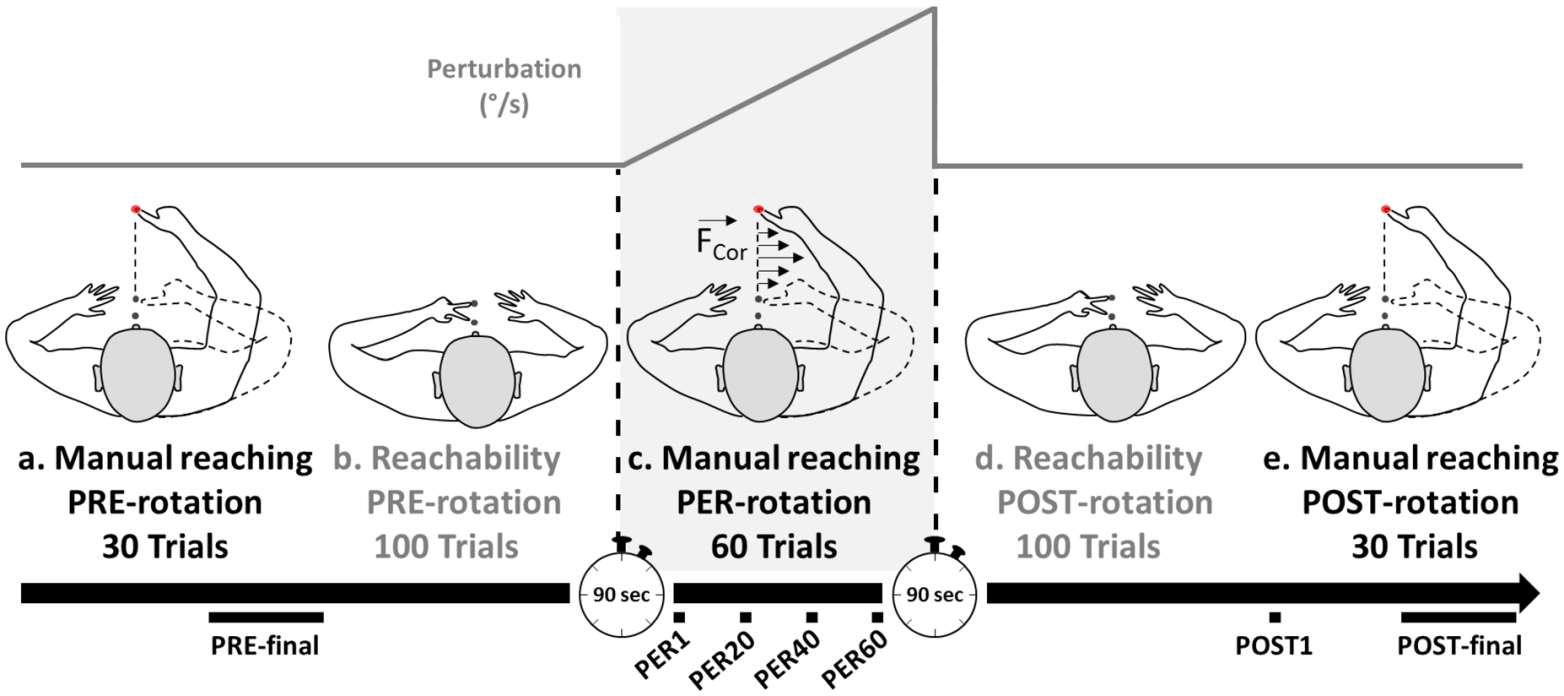
Illustration of the experimental procedure. Manual reaching performance was assessed before (**a**), during (**c**) and after (**e**) platform rotation, while reachability was estimated just before (**b**) and just after (**d**) platform rotation. Rotating speed was gradually increased by increments of 2°/s, from 0°/s to a maximum of 120°/s, over the series of 60 trials during the PER-rotation phase. The expected direction and magnitude of the velocity-dependent Coriolis force are illustrated on (**c**). At the bottom of the Figure, the manual reaching trials used for statistical analyses are shown under the black time arrow: the ten last trials before rotation (PRE-final), the first (PER1) and every twentieth trial (PER-20, 40 and 60) during rotation, and the first (POST1) and ten last trials (POST-final) after rotation.

All participants were familiarized with both tasks during a pre-experiment session. Then, the experiment involved the following five conditions, presented in successive blocks of trials (see Fig. 2).

- Manual reaching task/PRE-rotation (platform stationary). Participants executed a series of 30 reaching movements toward the visual target to determine baseline sensorimotor performance.
- Reachability judgment task/PRE-rotation (platform stationary). Participants performed a series of 100 reachability judgments (each of the 25 targets randomly presented four times) to determine baseline performance in reachability judgments.
- Manual reaching task/PER-rotation (platform rotating). The platform was gradually accelerated, counterclockwise, during the PER-rotation phase. As illustrated in Fig. 2, the angular velocity of the rotation was increased by 2°/s at every trial during the 60 trials performed by participants. These executed their first movement when the rotation speed was 2°/s and their last movement when the rotation speed was 120°/s (thus corresponding to the rotation speed used in our previous studies with an abrupt dynamic perturbation^26,33^). Consequently, the platform’s rotation generated gradually increasing Coriolis force (F_cor_) on the moving limb throughout the PER-rotation phase. After the 60th trial, the platform was decelerated progressively for 80 s (decrease of the rotation speed by 1.5°/s) until stationary.
- Reachability judgment task/POST-rotation (platform stationary). Participants performed a new series of 100 reachability judgments after the action control system may have been adapted to the platform rotation.
- Manual reaching task/POST-rotation (platform stationary). Participants ended the experiment by performing a series of 30 manual reaching movements toward the visual target.

A 90 s pause was included between the end of the platform rotation and the ensuing task, to allow the vestibular semi-circular canals to return to their resting discharge frequency^56^. For each block of trials, participants were instructed not to move their opposite arm (left arm during the manual reaching task, right arm during the reachability judgment task).

### Data recording and analysis

In the manual reaching task, the x, y and z coordinates of the marker on the right index fingertip were recorded and then analyzed via customized Matlab software (Mathworks, Natick, MA, USA). Raw data were low-pass filtered using a dual-pass, no-lag Butterworth (cut-off frequency: 8 Hz; order: 2). Velocity data were obtained from the filtered position data. As in Lefumat et al.^31^, movement onset was defined as the first time that hand velocity reached 3 cm/s and movement offset was defined as the first time that hand velocity dropped below 3 cm/s. These time landmarks were used to compute movement time.

Previous work showed that the Coriolis force mainly influences the directional control of movement^27,29,30,57^. We therefore computed initial movement direction, as given by the angle between the vector start position-to-target position and the vector start position-to-hand position at the time hand movement reached maximum velocity. We considered peak velocity to be of particular interest in the present study because it coincided with the maximum effect of the Coriolis force. Peak velocity was reached on average 268 ± 56 ms (mean ± SD) after movement onset. We also analyzed movement endpoint error, as given by the angle between the vector start position-to-target position and the vector start position-to-hand position at the end of the reaching movement^29,30^. In addition, we computed mean perpendicular deviation (mean PD) and maximum perpendicular deviation (max PD), respectively the average and the maximum distances between the hand and its orthogonal projection on the straight line linking the hand starting position and its ending position^58,59^. These commonly-used measures were chosen because, even though participants were not given specific instructions regarding the hand path, intended hand path is generally straight toward the target^60,61^. For all these variables, rightward trajectory deviations had positive values, and leftward deviations had negative values.

Sensorimotor adaptation to Coriolis force was characterized using comparisons similar to those of Lackner and DiZio^27^, Lefumat et al.^31^ and Leclere et al.^26,33^. Trial-by-trial analyses of initial direction, endpoint error, mean and maximum amplitude deviation were used to characterize adaptation to the perturbation. Data from the final ten trials in the PRE-rotation phase (labelled PRE-final) were averaged for each participant and used as baseline value. This baseline was then compared to the data for the first (PER1), the twentieth (PER20), the fortieth (PER40) and the sixtieth trial (PER60) performed during platform rotation. These PER-rotation trials were selected to analyze the time course of adaptation to the gradual increase of rotation speed. In addition, baseline was compared to the first (POST1) and the average of the final ten trials (POST-final) after rotation, to detect any after-effects.

Reachability judgments and the associated response times were analyzed. As in Bourgeois and Coello^23^ and Leclere et al.^26,33^, the estimated boundary of reachable space was determined using the logit regression model that best fitted the reachable/unreachable responses of the participants. Taking into account the 25 target positions, the model relied on the following equation: y = e^(α+βx)^/(1 + e^(α+βx)^), in which y was the participant’s response, x the distance between the target presented and the target representing the physical limit of reachability, and (− α/β) the value of x at which the transition from one type of response (reachable) to the other type of response (unreachable) occurred (the probability *p* associated with the logit function was 0.50 for both responses). This point of subjective equality (PSE) thus expresses the perceived boundary of reachable space used as a proxy of the limit of the peripersonal space representation. Positive values corresponded to rightward targets with respect to the boundary of the physically reachable space, or in other words to an overestimation of the peripersonal space boundary. In addition, we computed the discrimination threshold, defined as the distance between the target judged reachable at *p* = 0.50 (PSE) and the target judged reachable at *p* = 0.84^62^. The smaller the discrimination threshold, the more accurate the participants were in distinguishing between reachable and unreachable targets.

In the reachability judgment task, we defined response time (RT) as the time between stimulus onset and button press. We calculated the mean RT for each target position, which yielded 25 mean RTs per condition (PRE-rotation and POST-rotation) for each participant. Only individual RTs around the mean ± 2.5 standard deviations were included in the subsequent analysis (3.1% of the data were discarded in the PRE- and 3.3% in the POST-rotation condition). We then fitted RTs as a function of target position with a Gaussian regression model to estimate the distance at which maximum RT (RT max) occurred for each participant in each condition. Because the fit for four participants in each group yielded a maximum RT distance beyond the range of the targets, we considered these values as aberrant and excluded them from the analysis. RT analysis was thus conducted on ten participants in each group. Previous studies showed that typically, RT reaches a maximum for stimuli located at the boundary of the reachable space^10,23,63,64^. Finally, we calculated the Pearson coefficient (r) of the correlation between the target distance corresponding to the PSE and the target distance corresponding to the maximum RT on the Gaussian fit.

### Statistical analysis

To assess sensorimotor adaptation in the manual reaching task, we conducted a one-way analysis of variance on the factor Phase (PRE-final, PER1, PER20, PER40, PER60, POST1, POST-final) with repeated measures (RM-ANOVA). We conducted the same analysis to compare perceived reaching movements’ accuracy across the phases of the experiment, using a one-way analysis of variance on the factor Period (PRE-late, PER-early, PER-late, POST-early, POST-late) with repeated measures. When there was a significant main effect, a Tukey HSD post-hoc test was used for further analysis. We also determined for each participant whether data in the POST1 trial differed from the 95% Confidence Interval on PRE-final trajectories^65^. In the reachability judgment task, both perceived boundary of reachable space and discrimination threshold were compared between PRE- and POST-rotation conditions, using a t-test for related samples. Level of significance was 0.05 for all analyses. Normality of data distribution was verified in all experimental conditions, using the Kolmogorov–Smirnov method.

### Ethics approval

The study was approved by the institutional review board of the Institute of Movement Sciences and was performed in accordance with the Code of Ethics of the World Medical Association (1964 Declaration of Helsinki).

### Consent to participate

All participants gave their written informed consent prior to inclusion in the study.

### Consent for publication

All authors read and approved the final manuscript.

## Results

### Manual reaching task

#### Kinematic analysis

**Figure 3:**
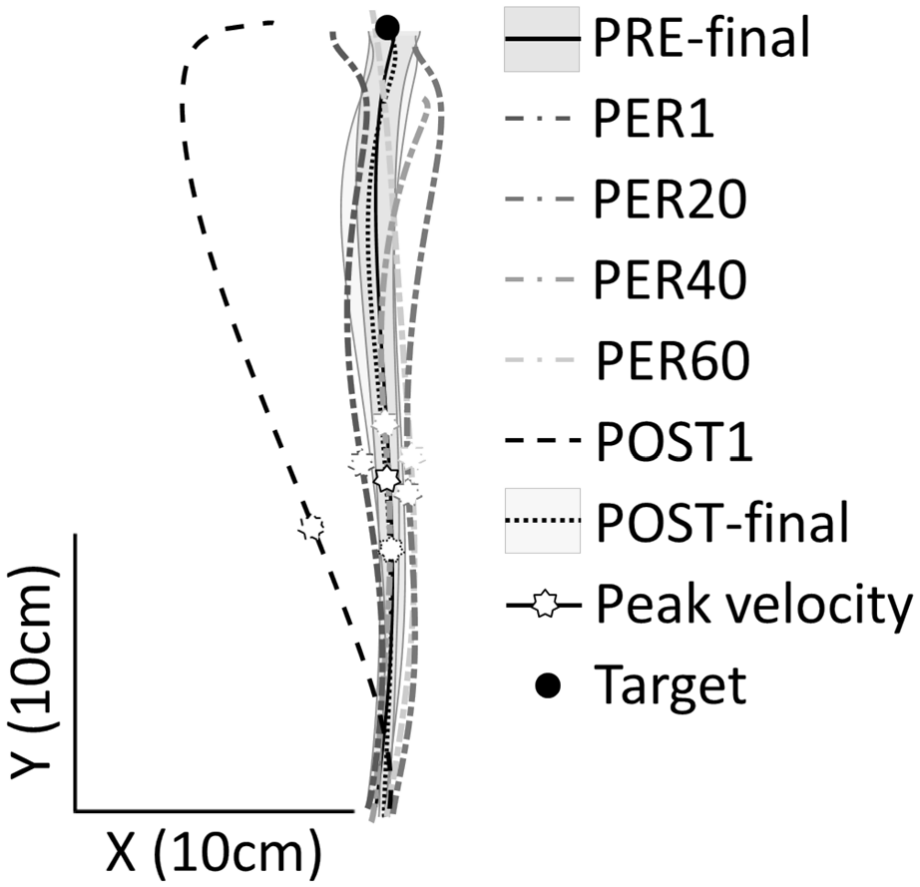
Top-view of manual reaching trajectories for a representative participant. Hand paths correspond to the mean trajectory of the ten last trials in PRE-rotation (PRE-final, black line), the first (PER1), the twentieth (PER20), the fortieth (PER40) and the sixtieth (PER60) trial in PER-rotation (gray dash-dotted lines), the first trial in POST-rotation (POST1, black dashed line) and the ten last trials in POST-rotation (POST-final, black dotted line). Standard deviations from the mean trajectories in PRE-final and POST-final are represented in gray areas. An after-effect was visible in the first trial following removal of the Coriolis force (POST1), which differed markedly from all other trials.

Baseline performance (PRE-final trials), was assessed before gradually increasing the rotation speed of the experimental platform and consequently introducing a new force field (PER trials). Participants’ reaching movements toward the target were nearly straight during baseline and did not differ much throughout the trials during platform rotation (see Fig. 3). However, a striking difference was observed in the first trial after the rotation stopped: movement trajectory was deflected to the left, i.e. opposite to the direction of the Coriolis force incrementally increased during the counter-clockwise rotation of the platform. Movement trajectory then recovered a straight path toward the target in a few trials, ultimately resembling that observed during baseline.

**Figure 4:**
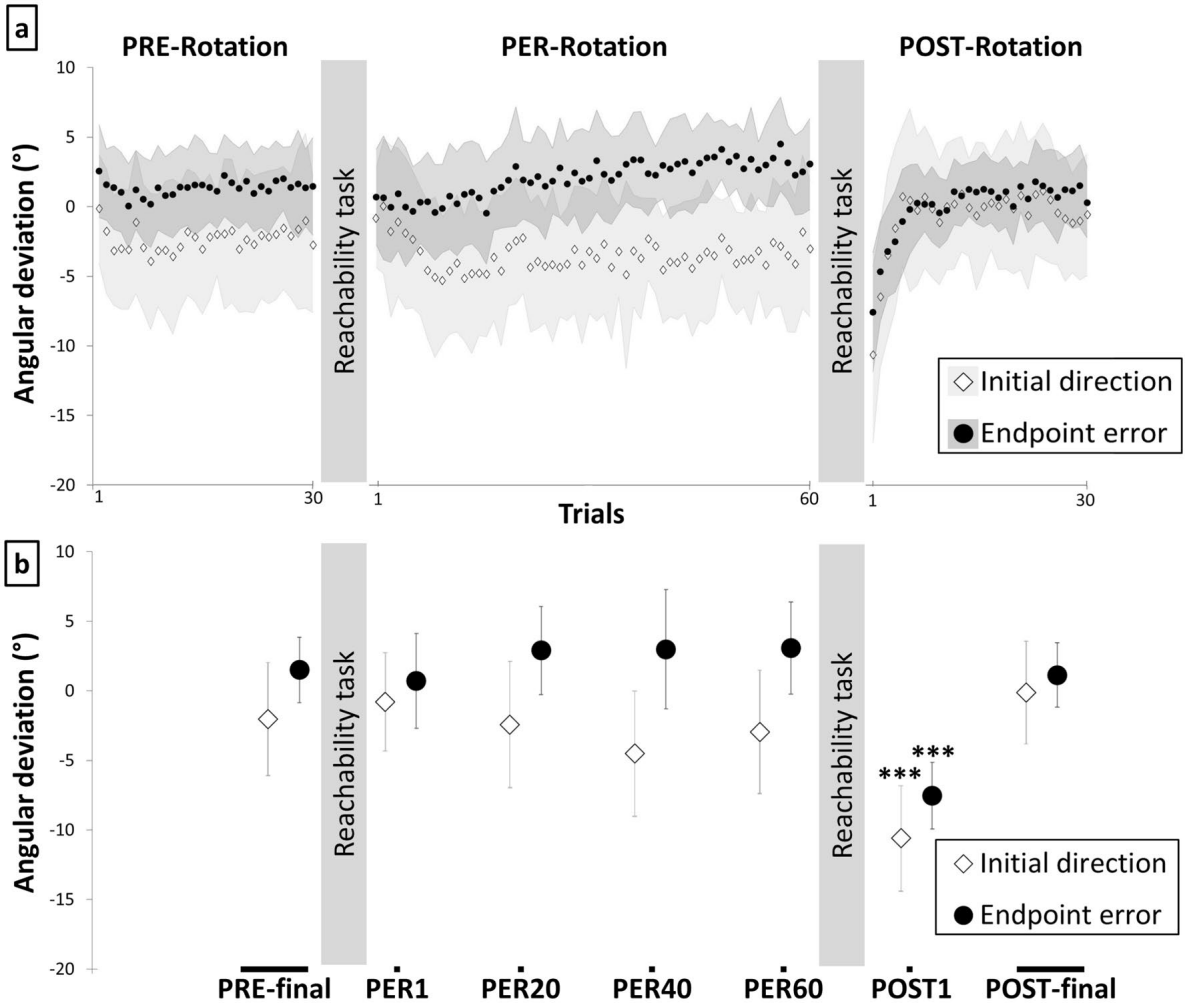
Sensorimotor adaptation to gradual force field perturbation. (**a**) Time course of mean angular deviation at peak velocity (initial direction) and final position (endpoint errors) across the experimental trials. (**b**) Time course of initial direction and endpoint error across selected trials. Stars indicate significant differences between one experimental condition and the baseline for each dependent variable (***p < 0.001).

The kinematic characteristics of the reaching movements were influenced by the experimental procedure, as revealed by a one-way RM-ANOVA with seven levels (PRE-final, PER1, PER20, PER40, PER60, POST1 and POST-final) showing a significant effect of Phase on initial direction of the movement (F(6, 84) = 13.53, p < 0.001, η^2^ = 0.49). HSD Tukey post-hoc comparison revealed that the initial direction of the POST1 trial was significantly deviated to the left (− 10.7 ± 6.2°) compared to PRE-final movement (− 2.1 ± 4.1°) and to any movement performed in the other phases (all p < 0.001), as shown in Fig. 4. Thus, the rightward Coriolis force generated during the counterclockwise rotation of the platform did not result in any significant rightward deviation of the reaching movement compared to baseline in the PER phase. However, an after-effect, i.e., a difference between the PRE-final and POST1 trial was observed overall. At the individual level, initial movement direction in the POST1 trial was considered to be deviated leftward for 14/15 participants as it was outside the 95% confidence interval computed for the last ten baseline trials.

A similar pattern of results, with a significant after-effect, was found for endpoint error (Fig. 4). RM-ANOVA revealed a significant effect of Phase (F(6, 84) = 31.86; p < 0.001, η^2^ = 0.69). POST1 endpoints were significantly deviated leftward (− 7.6 ± 4.3° which corresponded to 3.3 ± 1.9 cm) compared to PRE-final endpoint (1.4 ± 2.3°) and endpoints in all the other phases (all p < 0.001). Also, endpoint error in the POST1 trial was found to be leftward compared to the baseline in 13 out of the 15 participants and outside the 95% confidence interval computed for the last ten baseline trials. This analysis supports the idea of a significant after-effect following gradual adaptation to the altered gravito-inertial force field.

**Figure 5:**
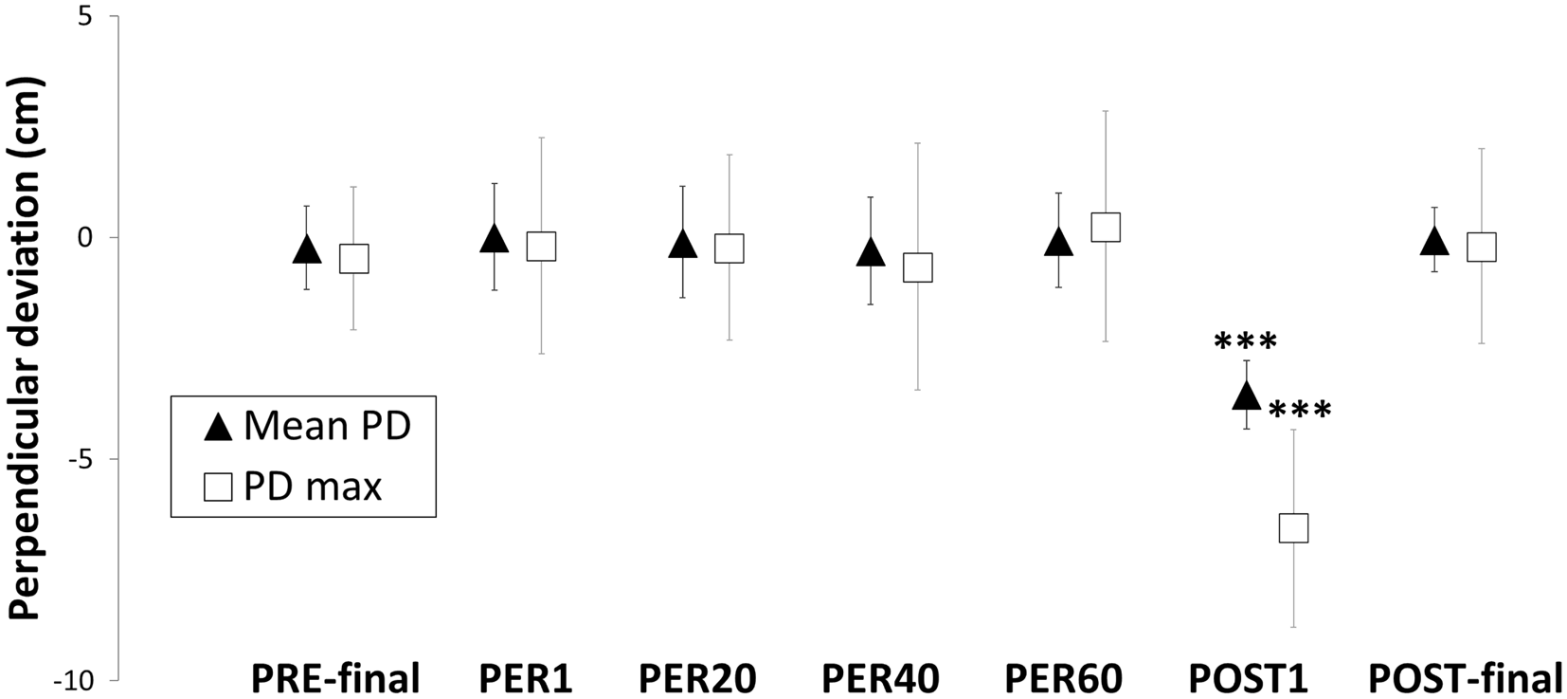
Perpendicular deviation of the trajectory from a straight-line linking start and end locations, as a function of the experimental Phase. Vertical bars represent standard deviation around the mean across participants. Stars indicate significant differences of mean and maximum perpendicular deviation in POST1 compared with all other phases (***p < 0.001).

RM-ANOVA revealed a significant effect of Phase on mean perpendicular deviation (F(6, 84) = 41.3; p < 0.001, η^2^ = 0.75), as illustrated in Fig. 5. Post-hoc tests showed that mean trajectories were significantly deviated to the left for POST1 (− 3.5 ± 1.3 cm) compared to PRE-final (− 0.2 ± 0.9 cm) and all the other phases (all p < 0.001). A similar pattern of result was found for maximum perpendicular deviation (F(6, 84) = 31.86; p < 0.001, η^2^ = 0.69). Post-hoc analysis revealed that maximum perpendicular deviation was leftward for POST1 (− 6.6 ± 2.1 cm) compared to PRE-final (− 0.5 ± 1.6 cm) and all other phases (all p < 0.001). Individual-level data analysis revealed leftward perpendicular mean deviation in POST1 trial for 15 out of the 15 participants, systematically outside the 95% confidence interval of the mean computed for the last ten baseline trials. The same pattern appeared for maximum perpendicular deviation. This analysis highlights differences in the POST1 trial with respect to baseline trials, pointing to a typical after-effect associated with sensorimotor adaptation.

RM-ANOVA on movement time revealed a main effect of Phase (F(6, 84) = 4.25, p < 0.001, η^2^ = 0.33) and post-hoc analysis revealed that movements lasted significantly longer in the POST1 trial (522 ± 136 ms) than in the PRE-final trials (431 ± 45 ms) and in all the other phases (all p < 0.001). This finding may be related to the final correction of the POST1 deviated trajectory that can be seen on Fig. 3. ANOVA revealed that peak velocity (mean = 160 ± 38 cm/s) did not significantly vary across experimental phases (F(6, 84) = 1.91, p = 0.09, η^2^ = 0.12).

### Reachability judgment task

**Figure 6:**
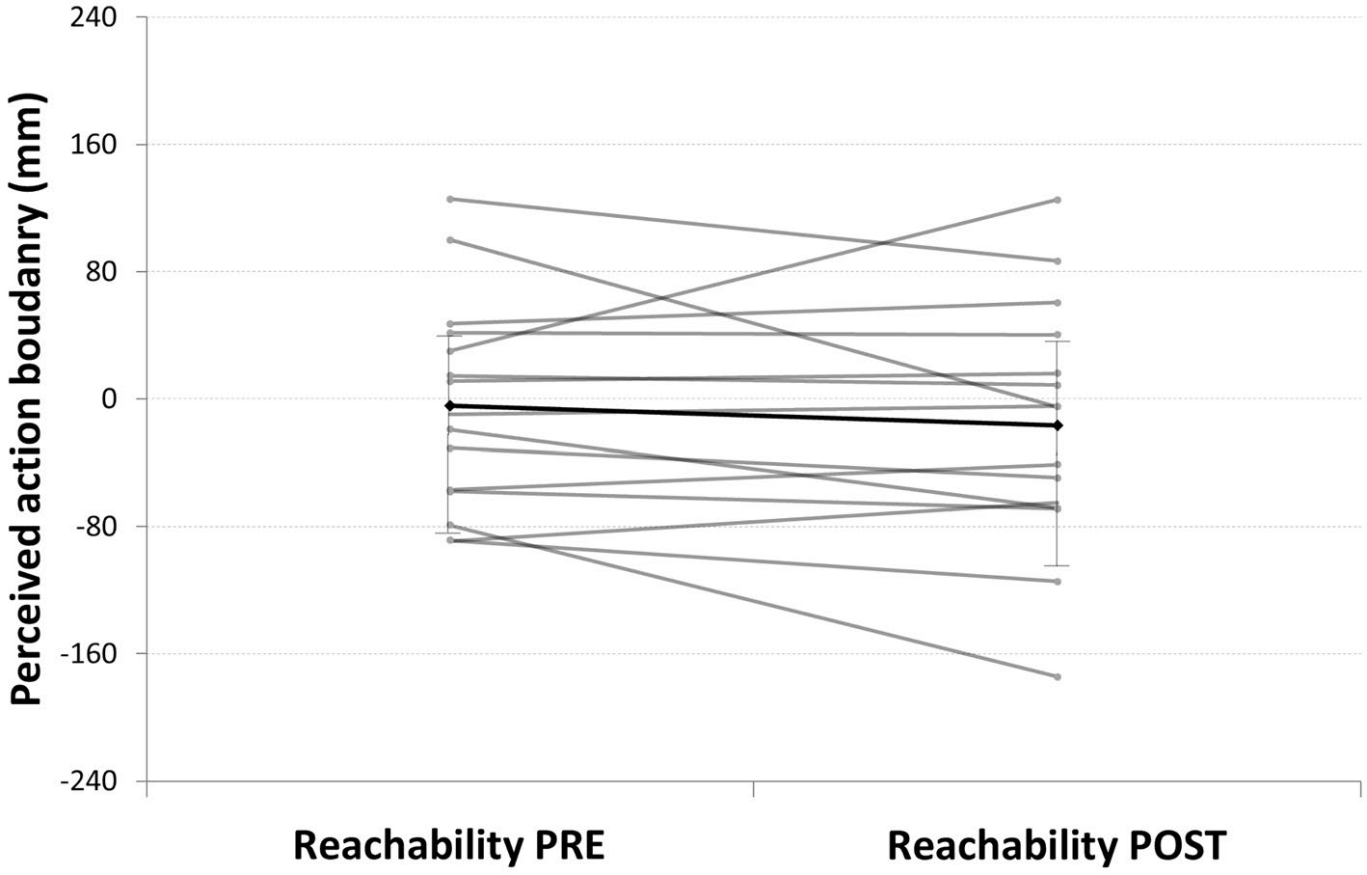
Perceived boundary of reachability in the PRE- and POST-test conditions, for every participant (grey) and on average (black). Vertical bars represent standard deviation of the mean.

A paired t-test showed no significant difference in perceived boundary of reachability between POST-rotation (− 17 mm ± 78 mm) and PRE-rotation phases (− 4 mm ± 66 mm; t(14) = 1.02; p = 0.32), as shown in Fig. 6. Moreover, a paired t-test showed no significant difference between PRE- (− 69 mm ± 29 mm) and POST-rotation conditions (− 73 mm ± 39 mm) in discrimination threshold (t(14) = 0.48; p = 0.64).

The lack of significant differences between the perceived boundary of reachability in the PRE- and POST-test conditions was not necessarily proof that gradual exposure to new limb dynamics did not influence reachability judgments. To gauge the strength of the null hypothesis (a null effect of gradual exposure to new limb dynamics on reachability judgments), we used Bayesian statistics^66,67^ with the JASP free software (https://jasp-stats.org). Using the Bayesian approach led to a BF01 score of 2.4, providing anecdotal evidence for the null hypothesis^66,67^.

**Figure 7:**
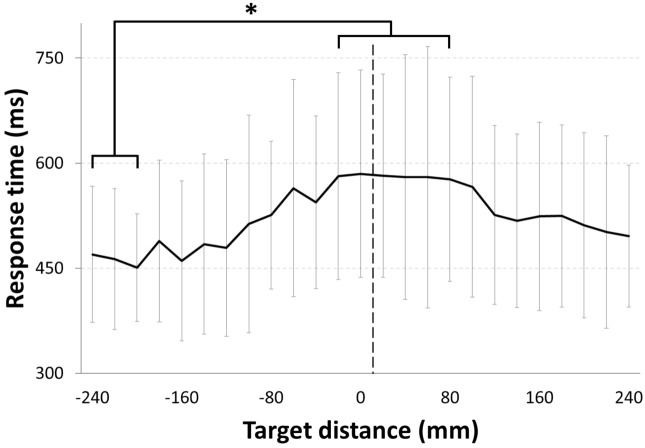
Response time for each target distance across participants; only main significant differences are represented. Target distance corresponding to the perceived boundary of reachability, averaged across participants, is represented by the vertical dotted line.

RM-ANOVA [25 Targets × 2 Conditions (PRE, POST)] on response time revealed a significant main effect of Target distance (F(24, 336) = 4.92, p < 0.001, η^2^ = 0.26) but no significant effect of Condition (mean PRE = 540 ± 135 ms, mean POST = 513 ± 144 ms; F(1, 14) = 4.46, p = 0.053, η^2^ = 0.24) or interaction between the two factors (F(24, 336) = 0.89, p = 0.62, η^2^ = 0.06). Significant differences in response time between target distances revealed by post-hoc analysis are shown in Fig. 7. In summary, response time for target − 240, − 220 and − 200 differed from targets − 20, + 20, + 40, + 60 and + 80 (p < 0.05) as well as target 0 (p < 0.01). Differences mainly involved shorter response times for targets positioned to the left, those which were closest to the participants and clearly reachable, than for those around the middle, which participants were uncertain of reaching.

We also fitted RT with a Gaussian distribution and computed the distance at which maximum RT was recorded for both PRE- and POST-rotation conditions. Linear regression analysis showed that the distance associated with maximum RT correlated with the distance associated with the perceived boundary of reachability, across participants, in both the PRE- (r = 0.75; p < 0.01) and POST-conditions (r = 0.92; p < 0.001). Moreover, a paired t-test showed no significant difference between distances associated with maximum RT in PRE- and POST-conditions (mean PRE = 540 ± 135 ms, mean POST = 513 ± 144 ms; t(10) = 1.05; p = 0.32). This additional analysis provides further support to the idea that gradual exposure to new limb dynamics did not significantly influence the perceived boundary of reachability.

### Complementary analysis with a control group exposed to an abrupt change of the Coriolis force

#### Reachability judgments

To strengthen our data, we decided to compare the current results (with 15 participants) to those obtained in a previous study^26^ (with 14 participants) in which participants were exposed to an abrupt change of the gravito-inertial force field. Given that we previously found a significant effect of an abruptly-introduced perturbation on reachability judgments, we included the reachability data from Leclere et al.^26^ in the statistical comparison, which was possible because of the similarity of the experimental procedure in the two studies. Reachability judgments were compared in two groups of participants (Gradual: present study, and Abrupt, Leclere et al.^26^) in both Pre- and Post-tests. A 2 × 2 (two groups: Abrupt and Gradual and two conditions: PRE-test and POST-test) repeated-measure ANOVA showed that there was a significant interaction between group and condition (F(1, 27) = 7.14, p = 0.13) on the PSE of the reachability judgement, as well as a significant effect of condition (F(1, 27) = 18.6, p < 0.001) but no significant effect of group (F(1, 27) = 0.82, p = 0.37). Post-hoc analysis of the interaction revealed that the only significant difference was between the PSE in PRE-rotation and POST-rotation in the group with an abrupt rotation (p < 0.001). A t-test on independent samples then showed that the shift in PSE between PRE- and POST-rotation was greater for the Abrupt group (N = 14; mean = − 52 ± 34 cm) than for the Gradual group (N = 15; mean = − 13 ± 48 cm; t(27) = 2.67, p = 0.01).

#### After-effects of Coriolis force adaptation

As no significant effect of the gradually-introduced perturbation was found on reachability judgments (whereas there is an effect of the abrupt perturbation^26^), we wanted to determine the robustness of the sensorimotor adaptation for the Gradual group of the present study and the Abrupt group of a previous study^26^. To do so, we analysed after-effects, and more specifically the time course of deadaptation in the manual reaching task. To this aim, we compared the evolution of endpoint errors in the two groups (Abrupt, Gradual) across the first ten trials of the POST-rotation reaching phase, and compared it to baseline (PRE-rotation). A 2 × 11 (2 groups of participants and 11 trials) repeated-measure ANOVA on endpoint errors of the last baseline trial and each of the first ten trials in POST-rotation for both groups of subjects in the Gradual experiment (present study) and Abrupt experiment^26^. The ANOVA showed that the Trial factor had a significant effect on endpoint errors (F(1, 10) = 39.9, p < 0.001). However, there was no significant effect of Group (F(1, 1) = 2.9, p = 0.1) and no significant interaction (F(1, 10) = 1.4, p = 0.19). A post-hoc analysis (HSD Tukey) of the Trial main effect showed that endpoint error at baseline (mean = 1.8 ± 3.0°) differed from that at the first four POST-rotation trials (mean POST1 = − 8.1 ± 4.8°; mean POST2 = − 4.3 ± 3.9°; mean POST3 = − 2.0 ± 3.4; mean POST4 = − 1.3 ± 3.8°; all p < 0.001). For trials POST 5–10, there was no significant difference with baseline. This statistical analysis indicates that it took on average five trials for participants to deadapt to the perturbation, regardless of the way the perturbation was introduced (gradually or abruptly).

To summarize, whereas no differences were found in time-course of sensorimotor adaptation between the two groups, the present analysis shows that the shift in the boundary of reachability was significant in the abrupt group but not in the gradual group.

## Discussion

Previous studies showed that the representation of the peripersonal space is modified following abrupt exposure to new limb dynamics^26,33^. Such abrupt exposure was found to lead to typical sensorimotor adaptation^27,29,31,32,35^ as well as to altered reachability judgments. These previous findings were consistent with a systematic contraction of the representation of the peripersonal space in response to large motor errors induced by a modification of the Coriolis force when introducing a new gravito-inertial force field. The aim of the present study was to determine whether the representation of the peripersonal space is also modified when sensorimotor adaptation occurs in the absence of large motor errors. To do so, we assessed the representation of the peripersonal space, using the same experimental procedure as in our previous studies^26,33^, before and after exposure to a gradual perturbation of limb dynamics through a rotating device that was expected to induce cumulative motor errors of small amplitude. We found a significant motor after-effect following adaptation to the gradually-introduced force field, but reachability judgments were not significantly affected. This suggests some degree of independence between the processes underlying sensorimotor control and those contributing to the representation of the peripersonal space. On the other hand, the influence of an abruptly-introduced force field on both reachability and reaching tasks^26^ leads us to speculate that cognitive mechanisms associated to the conscious perception of perturbations could mediate the link between perceptual and action control mechanisms.

### Sensorimotor adaptation to a new gravito-inertial force field

In the present study, as rotation gradually accelerated, the Coriolis force progressively increased throughout the successive trials. This gradual modification of the force field led to no significant change in movement kinematics, although it was detected by the sensorimotor system as evidenced by the substantial after-effect observed for each participant after the rotation phase. Movement trajectories during exposure to the force perturbation were indeed quite similar to baseline, with no significant differences found between baseline (PRE-rotation) and PER-rotation trajectories on several kinematic parameters. The experiment was done in a dark room and state estimates of actual limb position and movement had to be based on proprioception (from muscles, tendons, joints and skin^44^). To the best of our knowledge, proprioceptively-based adaptation to gradual changes in Coriolis force had never been demonstrated before and the present study provides the first evidence. Current theories suggest that the slight mismatch between intended reach and actual reach during the exposure phase induced a slow process of sensorimotor adaptation^49,53,68^. In other words, small sensory prediction errors may have resulted in a gradual modification of the motor commands sent to the muscles to reach toward the visual target, taking into account the modified force field. These findings are consistent with those of previous studies in which a large after-effect is observed even when movement kinematics are only slightly modified during exposure to a gradual perturbation, for instance with a robotic perturbation of arm dynamics^37,48,49^.

Previous investigations of force-field adaptation reported large motor errors when suddenly exposed to abrupt change in the gravito-inertial force field^27,30,32,57^. In the present study, the change in force field was gradual and likely led to small motor errors throughout the exposure. Despite the disparity in motor errors induced by abrupt versus gradual force field alteration, after-effects did not significantly differ: for example, maximum perpendicular deviation was around 8 cm in both the present study and Leclere et al.^26^. In fact, in a complementary analysis involving a group exposed to an abrupt change of the Coriolis force^26^, we compared the temporal decay of after-effects between gradual and abrupt groups. Both groups recovered a reach precision close to baseline after four trials during POST-rotation and endpoint errors vanished with the same temporal pattern whatever the type of perturbation. Overall, adaptation to the new gravito-inertial force field appeared, in the present study, to have a similar effect on the sensorimotor system as in previous studies^27,36,37,52,59^, even though the mechanisms underlying sensorimotor adaptation might differ according to whether the perturbation is introduced gradually or abruptly^50,69^.

### Representation of the peripersonal space

Our results indicate that, despite the substantial sensorimotor adaptation, the representation of the peripersonal space did not significantly change after gradual exposure to the modified force field. This finding contrasts with the previous finding of a systematic contraction of the representation of the peripersonal space when a new force field was abruptly experienced^26,33^. These different results suggest that the previously observed contraction of the peripersonal space representation might be related to the abruptness of exposure to the limb dynamics perturbation rather than to sensorimotor adaptation per se. We hypothesize that large motor errors, presumably consciously detected, could lie behind such contraction.

One possibility is that the conscious perception of large motor errors following abrupt change of the gravito-inertial force field led to a decrease in the estimated reliability of the sensorimotor system, resulting in a conservative strategy regarding object reachability. As the peripersonal space plays a crucial role in the control of action as well as in the protection of the body from external hazard^70–73^, its representation may shrink when it is required to minimize the risk of motor errors and to maximize the efficiency of the sensorimotor system. Most studies on sensorimotor adaptation assumed that small motor errors induced by a gradually increased perturbation precluded clear awareness of the perturbation and the adaptation^37,39,48,53,59,74^. We suggest that under gradual perturbation of the force field, the ensuing motor errors may be too small to trigger such a conservative strategy, in relation to the representation of the peripersonal space. This interpretation is consistent with the idea that the representation of the peripersonal space depends on cognitive factors associated with the perception of what is reachable in the near-body space, taking into account reward prospects^75^. However, further work is necessary to determine whether different methods would support the present interpretation that gradual exposure to new limb dynamics does not influence reachability judgments.

## Conclusion

In the present study, we observed that gradual exposure to a modified gravito-inertial force field resulted in systematic sensorimotor adaptation but did not affect the representation of peripersonal space. While several studies showed a link between alteration of the motor system and the representation of the peripersonal space, our study suggests that the processes contributing to motor performances and those contributing to the representation of peripersonal space could be updated independently. Further work would be necessary to test the hypothesis that the conscious processes associated with sensorimotor adaptation may play a role in the contribution of the motor system to the representation of the peripersonal space.

## Data Availability

Data are available upon reasonable request.

## Code availability

Code is available upon reasonable request.
